# How to Increase Reach and Adherence of Web-Based Interventions: A Design Research Viewpoint

**DOI:** 10.2196/jmir.4201

**Published:** 2015-07-10

**Authors:** Geke DS Ludden, Thomas JL van Rompay, Saskia M Kelders, Julia EWC van Gemert-Pijnen

**Affiliations:** ^1^Department of DesignFaculty of Engineering TechnologyUniversity of TwenteEnschedeNetherlands; ^2^Center for eHealth Research and Disease ManagementDepartment of Psychology, Health and TechnologyUniversity of TwenteEnschedeNetherlands

**Keywords:** Web-based interventions, adherence, design for well-being, metaphors, personalization, ambient information

## Abstract

Nowadays, technology is increasingly used to increase people’s well-being. For example, many mobile and Web-based apps have been developed that can support people to become mentally fit or to manage their daily diet. However, analyses of current Web-based interventions show that many systems are only used by a specific group of users (eg, women, highly educated), and that even they often do not persist and drop out as the intervention unfolds. In this paper, we assess the impact of design features of Web-based interventions on reach and adherence and conclude that the power that design can have has not been used to its full potential. We propose looking at design research as a source of inspiration for new (to the field) design approaches. The paper goes on to specify and discuss three of these approaches: personalization, ambient information, and use of metaphors. Central to our viewpoint is the role of positive affect triggered by well-designed persuasive features to boost adherence and well-being. Finally, we discuss the future of persuasive eHealth interventions and suggest avenues for follow-up research.

## Introduction

### Health Today

Our society faces severe problems when it comes to securing health for the public at large. First of all, unhealthy lifestyles, leading to diseases like obesity, are responsible for an unrelenting rise in health care costs. Secondly, long-term diseases like chronic obstructive pulmonary disease (COPD) and diabetes require life-long management of illness and attention from care professionals for a growing group of people. And thirdly, an aging population demands more intensive care. Next to (predominantly) physical health problems, a substantial number of people suffer from mental illnesses like burnout and stress disorders that likewise affect physical health in the long run.

Technology can play a role in stimulating people to take responsibility for their own health and well-being. Before we give examples of how technology can support people in taking this responsibility we will explain the relationship between health and well-being, and define the term well-being as we will use it throughout this paper.

### Well-Being, Health, and Technology

Several researchers have found that there is a two-way relationship between well-being and health: health influences well-being and well-being itself influences health. Both physical health and mental health influence well-being [1,2] and there are a number of correlations between well-being and physical health outcomes, such as improved immune system response, higher pain tolerance, increased longevity, cardiovascular health, slower disease progression, and reproductive health [3,4]. A meta-analysis even showed that emotional well-being predicts long-term prognosis of physical illness [5].

Different perspectives on the term well-being can be differentiated. Following Seligman’s [6] well-being theory, we see well-being as a multi-componential concept comprising positive emotions, engagement, meaning, positive relationships, and accomplishment. We will further elaborate on how these different elements can contribute to well-being in the following sections.

As said, technology can play an important role in helping people to take responsibility for—and, for that matter, to give them more control over—their own health and well-being; and it can do so in several ways. Firstly, technology can play a role in helping people to manage their disease(s). Web-based platforms offer a way to communicate with health professionals and/or to manage health conditions. For example, people who suffer from diabetes can make use of apps that help them understand what makes blood sugar levels rise and fall, and thus how to control such fluctuations. Next to this, medical technology has been introduced that supports people to take physiological measurements at home and automatically send these to a medical professional. For example, people who need regular blood pressure monitoring can now use certified devices at home. For many people, this is a more acceptable way of continuous regular monitoring [7]. Furthermore, technology can provide health treatment; in the area of mental health, a variety of Web-based interventions have been shown to be effective in providing (guided) self-help therapy to reduce complaints, as in Barak et al [8], or to lead a more flourishing life. Chitarro and Vianello [9] explored how technology can support mindfulness exercises and found that technology-supported mindfulness may be more effective than traditional (paper-based) exercises. Technology can also play a role in helping people to lead a healthier lifestyle in order to prevent diseases. In this area, devices that track physical behavior and provide feedback are often combined with a range of mobile and Web-based interventions that help people to lose weight, to be more physically active, or to adopt a healthier diet.

### Reach and Adherence: The Role of Design

The developments and examples above show that technology and, more specifically, Web-based interventions have a huge potential in (preventive) health and well-being. However, Web-based interventions often suffer from nonadherence: many people do not follow a treatment online as it was intended by the therapist. A systematic review of 83 Web-based interventions on lifestyle, chronic disease, and mental health found that, on average, around 50% of the participants adhere fully to an intervention [10]. This seems to reduce treatment effectiveness [11]. On top of this, most interventions reach a limited group only, while aiming for a broad audience. This does not have to be a problem; a strength of Web-based interventions may be that they can reach groups that are harder to reach by regular, face-to-face interventions, as seen in Postel et al [12] where e-therapy for problem drinking reached higher-educated females, while this group is underrepresented in regular care. However, several researchers have shown that current Web-based interventions often reach higher-educated women, as seen in several studies [13-17], while they fail to reach other groups in society. In some cases, these findings may have been influenced by the topic of the intervention under study, for example, the studies of Rothert et al [13], Binks and van Mierlo [14], and Kelders et al [15] were all aimed at weight management, a topic that may be of particular interest to higher-educated women. However, the overrepresentation of this specific group was also found for areas like alcohol abuse [12], depression [16], and smoking [17]; topics that seem at least relevant to many more groups in society. It seems there is something about current Web-based interventions themselves that attracts one specific group, and that in spite of their large potential, Web-based interventions miss out on helping the public at large. Kelders [10] argues that the design of interventions is an important factor for adherence and shows that there are persuasive features (eg, dialogue support, reminders and praise) when implemented in Web-based interventions that predict higher adherence. Moreover, in their review study on the effectiveness of eHealth interventions for physical activity and dietary behavior change, Norman et al [18] claim that interventions that feature interactive technologies in particular need to be refined to fully live up to their potential. In line with this claim, several researchers have proposed that the affective experience of persuasive technologies is the key to their effectiveness [19,20]. They reasoned that if the experience of, for example, a Web-based intervention is a pleasant one, people are inclined to keep using the intervention or to use the intervention again at a later point in time. In other words, a better design that is not only functionally effective but also desirable, compelling, and delightful could improve acceptance of, and adherence to, Web-based interventions. Introducing a "better" design to increase the affective experience of using a Web-based intervention could have a positive effect on well-being because it triggers positive emotions (the first element abbreviated as "P" in positive emotions, engagement, positive relationships, meaning, and accomplishment [PERMA]). However, this is a rather limited view on design’s impact on well-being. In an effort to further specify how design can contribute to well-being, Pohlmeyer [21] constructed a design well-being matrix that specifies how design can have an impact on the different elements in Seligman’s [6] well-being theory. As described, in this theory Seligman distinguishes five elements that can each contribute to a general feeling of well-being: positive emotions, engagement, positive relationships, meaning, and accomplishment. Pohlmeyer argues that design can take different roles that designers can use to intentionally design for well-being: source, symbol, enablement, and support. As examples, she describes how a product such as Paro the therapeutic robot seal, which is being used in care homes with dementia patients, can be the direct *source* of a *relationship* (R in PERMA) and how products can have an indirect effect on elements of PERMA by serving as a *symbol*. Think, for example, of a wedding ring (symbol of a relationship) or of a trophy (symbolizing achievement).

### Influencing Well-Being via Two Routes

**Figure 1:**
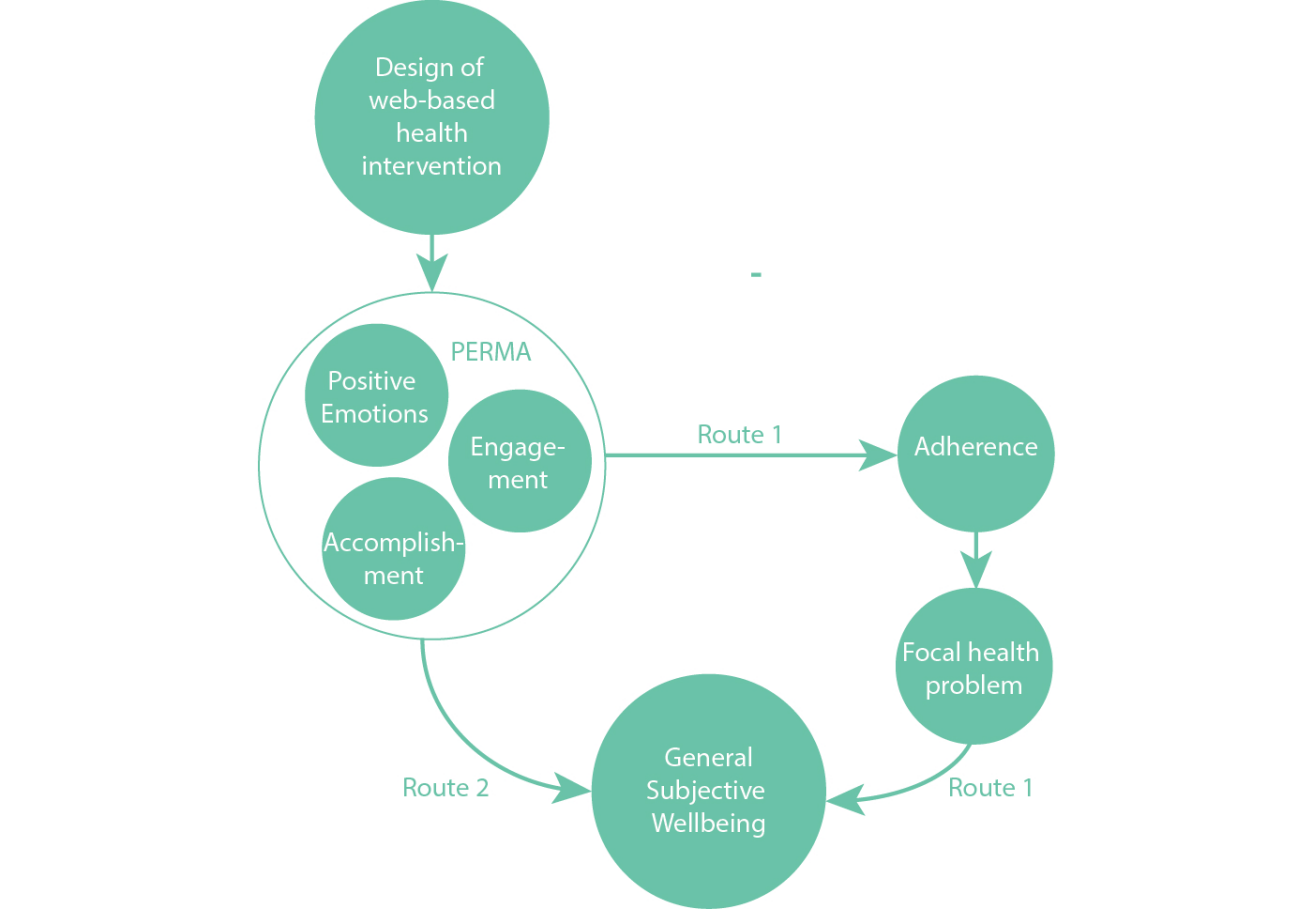
Schematic representation of how the design of a Web-based intervention can influence general subjective well-being following two different routes. Route 1 indicates the impact of design on adherence and thus on the focal health problem. Route 2 indicates how overall well-being is stimulated by elements of PERMA.

In the context of the design of a Web-based intervention, we would like to argue that design can have a positive effect on well-being following two different routes. First of all, a design aimed at a positive user experience by inducing, for example, positive emotion and/or engagement (P and E in PERMA) could positively influence well-being. Secondly, well-being during use could have an indirect effect on overall well-being because it can have a positive effect on adherence (ie, using the intervention as intended by the therapist). And better adherence to a Web-based intervention eventually has a positive effect on health, and thus on well-being. Next to this, successfully using the Web-based intervention could lead to a feeling of accomplishment (A in PERMA), again directly positively influencing well-being. Figure 1 shows how technology-supported health interventions can influence overall well-being via two routes.

The idea here is that, whereas the first route has a direct impact on the specific health problem (ie, people adhere and thus can manage and cure the focal health problem), the second route stimulates overall well-being by promoting states of mind that are conducive to a general feeling of well-being.

In this paper, our aim is not to test the relationships suggested in Figure 1. Rather, it should serve as an illustration that underlines the importance and the power of design. In the following sections, we will present a design research viewpoint to Web-based interventions and discuss new—to the field of designing Web-based interventions—design tools and approaches. We will explain and discuss how implementing these approaches could have a positive effect on reaching target groups and on adherence. To start, we will zoom in on Web-based health interventions and give a short overview of the types that are currently used. Next, we will illustrate with three examples how design could influence the experience of these interventions and contribute to an increased level of well-being when using such interventions. Finally, we discuss the future of Web-based health interventions and other technology-based, well-being interventions.

## Web-Based Interventions

### Overview

Web-based interventions can be categorized within the second generation of health interventions that make use of technology to transfer information [18]. Third generation interventions include mobile and remote devices such as mobile phones. We will include this third generation in some of our examples and in the discussion, but we will for now focus on the design of Web-based interventions because these form a widely used platform to implement technology-based health interventions.

### Current Design of Web-Based Interventions

Design of Web-based interventions is often content driven and text based, and aimed at education, information, and goal setting via modules or programs that have to be followed in a strict or fixed order. In many cases, content for a Web-based intervention was developed earlier as a fixed (offline) program that should later be “placed” on the website or mobile phone app. This often results in a text-driven, handout app, with the look and feel of a self-help book. Research on nonadherence indicates that reasons for dropout relate to dissatisfaction with the intervention, mismatch of goals of the intervention and those of the users, and low flexibility to adjust to different situations and user characteristics [15,22,23]. As a consequence, irritation rather than positive emotion (P in PERMA), and frustration rather than feelings of accomplishment (A in PERMA) could be triggered. Seen in this light, it should come as no surprise that low adherence is inherent to such interventions. However, it is precisely this "adherence" factor that is so essential for a positive effect of an intervention on health and well-being to transpire [11].

**Figure 2:**
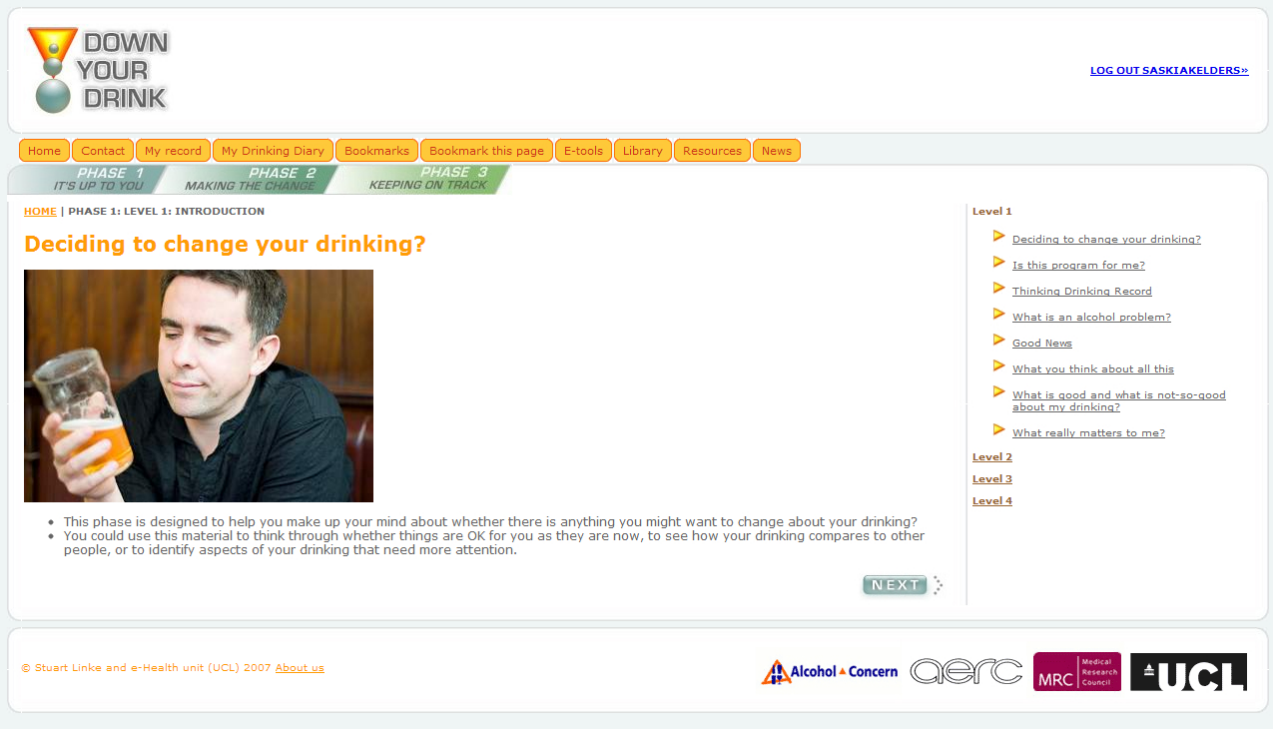
The Web-based intervention, Down Your Drink.

To further illustrate how low adherence may follow from ill-suited design, consider the Web-based intervention in Figure 2, aimed at reducing alcohol consumption. This intervention consists of six lessons, which are intended to be completed during 6 weeks [24,25]. Although some of the capabilities of technology are employed (eg, the provision of tailored feedback), the intervention is text based and technology seems to be mostly used as a medium to deliver the text. An analysis revealed that only 16.5% (of the first 10,000 registered users of the intervention) completed all 6 weeks of this intervention. A reason for nonadherence may well relate to a lack of attention to design and the resulting dissatisfying user experience. Limited reach of mobile and Web-based interventions could be explained by the fact that many of these interventions are aimed at a wide demographic group (eg, men and women, young and old, highly and less educated). Think, for example, of the range of track-and-trace systems aimed at keeping a healthy diet, losing weight, or increasing physical activity (eg, Lifesum, LosIt, MyFitnessPal). However, as we have argued in our introduction, most interventions reach a limited group only, mainly highly educated women—see, for example, Kelders et al [15]. In one way, this is a good thing, since this group in particular can be hard to reach with traditional treatment. However, this selective reach is not intended and, in many cases, seems to strengthen the "inverse care and information law" (ie, people in urgent need for care are the ones who are least likely to receive care [26,27]).

**Figure 3:**
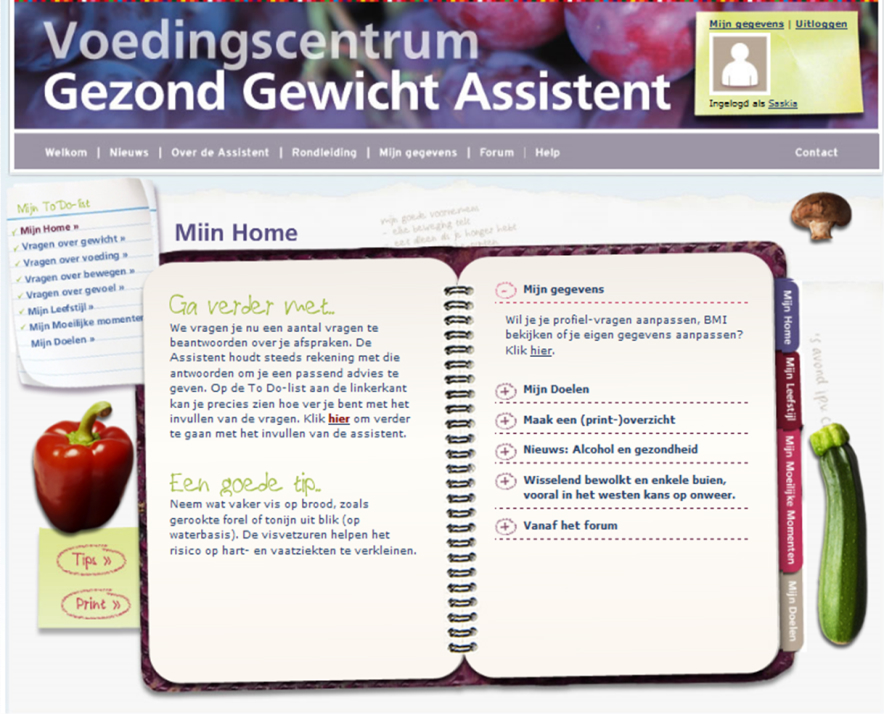
The Web-based intervention, Healthy Weight Assistant.

Consider another example of an intervention facing problems with reach and adherence (see Figure 3). The Healthy Weight Assistant is aimed at adults with healthy weight or who are slightly overweight. However, users were mainly female and highly educated [15]. Moreover, adherence to this intervention was as low as 3%, which the authors attribute to a mismatch between the goals of the intervention—long-term weight management—and the goals of the users—gain insight into their behaviors. This mismatch may have led to the low satisfaction and adherence found in the study.

### Effect of Design on Adherence

There have been multiple attempts to investigate the influence of characteristics of interventions on adherence. Two qualitative systematic reviews investigated the influence of specific characteristics on adherence and indicated that increased personal relevance, an individualized approach (eg, involving tailored advice and feedback), and including clinicians are promising avenues [28]. In terms of PERMA, these characteristics are most directly related to engagement. Perceived relevance promotes engagement (E) and positive relationships (R), furthermore, including clinicians introduces a relationship (R). Additionally, peer support, counselor support, email/phone contact with visitors, and updates of the intervention website—direct means to trigger relationship (R) perceptions—resulted in improved reach [29]. Note that introducing such personal elements to trigger relationships is a costly and possibly time-consuming way to improve adherence. From a design point of view, it would be interesting to introduce relationship perceptions through design elements (eg, by color, layout, and typeface) connoting relationship-related meanings, such as involved, empathic, and dedicated. Alternatively, one could think of introducing relationships with digital avatars.

A third systematic review investigated the influence of persuasive technology features and characteristics of Web-based interventions [10], demonstrating the importance of increased interaction with a counselor, more frequent updates, and more extensive employment of dialogue support. This latter aspect in particular seems to provide a starting point for designing for well-being. The concept of dialogue support stems from the persuasive systems design model [30] and refers to supporting the interaction between user and system to facilitate progress toward goal fulfillment. These principles include social and cognitive prompts, such as praise, rewards, reminders, and suggestions.

There have also been studies that investigate the influence of one or more features on adherence in a single intervention. For example, telephone reminders have been shown to increase adherence to a Web-based treatment of social phobia without clinician guidance [31], and emailed messages have been shown to lead to a modest increase in usage of a disease prevention website by some adults [32]. Although these studies suggest a relationship between the design of an intervention and adherence, only a few of these interventions were actually, and purposefully, designed with adherence in mind [10]. This suggests that adapting Web-based interventions to promote adherence is done in an ad hoc manner or is considered as a task for the counselor involved in the intervention. Studies that *have* tried to design for adherence have mainly focused on adding or adjusting features without explicitly considering the user experience as a means to improve reach and adherence.

## Design Approaches to Increase Reach and Adherence

### Overview

This short overview and discussion of Web-based interventions showed that while key works in the field are beginning to address reach and adherence challenges through increased interactivity and some possibilities for personalization, using a design strategy that effectively increases reach and/or adherence remains challenging. At the same time, technology offers many opportunities here; for example, it enables different means of communication simultaneously (eg, text, speech, video, and graphics) and provides access to situations and settings (eg, the bathroom) in which human persuaders would not be allowed in, or have no access to (eg, sensors in clothes) [33,19]. Therefore, we argue that for the design of Web-based interventions, it is worthwhile to look at how the field of design research is incorporating design strategies to design technology for well-being that delights people during use.

While seeking inspiration in the field of design research, it must be mentioned that a mere focus on expressive, creative design may result in technology that is likewise hard to understand and that has no fit with users’ mental models. As such, creative designs can overshoot the mark because users do not experience such designs as supportive. On the other hand, the more traditional approach of carrying out a needs-and-demand assessment provides information about some functionalities of a design, but may not reveal that much about motivational cues for adherence and experience. Therefore, we propose to use other design approaches that go beyond both the mere "design creativity-based approaches" as well as requirements engineering.

In the following sections, we discuss three design strategies—personalization, use of ambient information, and use of metaphors—that have been particularly well explored in design research as strategies to attract and involve people. These can have a positive effect on how someone experiences a Web-based intervention, thereby increasing both well-being during use and overall well-being. Cooperation among experts from different disciplines and discussion along these lines may help in creating technology-based interventions that contribute to well-being by targeting the three dimensions of happiness as discussed by Seligman [34]: the pleasant life, the good life, and the meaningful life (or positive affect, flow, and meaning). Thus, technology should render Web-based apps more appealing, engaging, and fun (positive affect), stimulate flow by paving the way for smooth and intuitive interaction—for example, by providing ambient rather than in-your-face or, reversely, completely hidden information—and create meaning by giving users a sense of control, direction, and purpose.

### Design Approach I: Personalization

In different application fields, researchers have shown that personalization of systems' functionalities and content can improve people’s satisfaction with services and can increase users’ efficiency and convenience. In PERMA terms, we would like to argue that personalization, foremost, promotes engagement (E) as it involves users and heightens personal relevance. Halko and Kientz [35] revealed significant relationships between personality and different types of persuasive technologies. Kaptein et al [36] measured susceptibility to persuasion and studied effects of tailored, persuasive text messages to reduce snacking. Results showed that tailored messages lead to a higher decrease in snacking consumption. In line with these findings, Lee et al [37] demonstrated that in the design of persuasive technologies for healthy eating, planning strategies worked differently depending on whether the participants had already adopted healthy dietary lifestyles.

**Figure 4:**
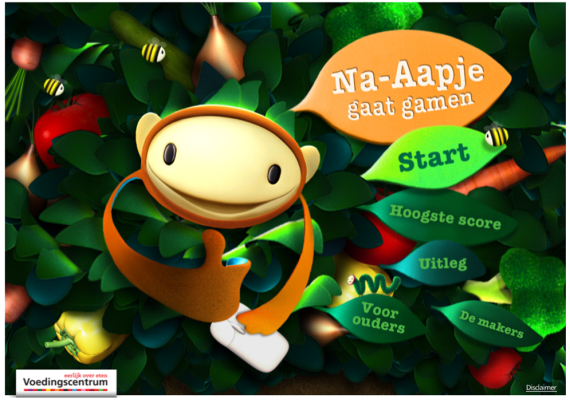
The Web-based intervention, Na-Aapje (The Netherlands Nutrition Centre).

These examples suggest that effects of design on adherence vary depending on users’ personal needs and that personalization is therefore important, as it enables connecting to specific needs of different people. There are three ways to personalize a Web-based intervention and create larger reach. The first way is in line with the examples mentioned above and involves tailoring of messages or (persuasive) approach. Secondly, a designer can set out to design for a specific target group. To do this effectively, knowing what a specific group needs and wants, and what motivates them is essential. A designer can use this knowledge to inspire and direct the creative processes leading to an intervention that creates engagement for a specific group of people. An excellent example of a Web-based intervention that is aimed at a specific target group and was designed with the needs, habits, and desires of this target group in mind is the game Na-Aapje that was released by the Dutch Voedingscentrum (The Netherlands Nutrition Centre) a few years ago. Na-Aapje (loosely translated as little copy-cat) is a children’s game that is designed to raise children’s awareness of fruits and vegetables as healthy diet choices. The monkey in the game has to collect food items resulting in a higher overall score if many fruits and vegetables are collected (see Figure 4).

In this way, Na-Aapje links a, perhaps, not pleasurable (ie, eating fruits and vegetables) but necessary activity for its user—the child—to a more pleasurable one. Many children like to play computer games and by connecting to their preferred activities, adherence to this intervention (and awareness of the importance of eating fruits and vegetables) is probably increased.

A third way to personalize a Web-based intervention could be to design an intervention in such a way that it can be changed or set up at the start to match user preferences. This could be done at both the content level and at the system (design) level. From a psychological point of view, this user-controlled type of personalization is particularly interesting as it gives users a sense of control or dominance which may contribute to general well-being. For instance, in Mehrabian and Russell’s [38] framework addressing emotional experiences in environmental settings, dominance is, next to pleasure and arousal, considered an essential factor in explaining approach-avoidance behavior, with higher degrees of control generally related to increases in approach behaviors—a notion similar to adherence in the online context—and hence, increased well-being.

Of further interest in this context is Averill’s [39] discussion of "decisional control," defined as the degree to which a specific action results from choice among various alternatives. For instance, a much-cited study by Mills and Krantz [40] showed that allowing blood donors a choice over which arm to use had a positive effect on donors’ experiences in blood transfusion centers. Extending these findings to consumer settings, Hui and Bateson [41] showed that conditions of crowding—generally related to decreases in control as other people in store environments lengthen shopping time and may block access to aisles and products therein—sorted fewer negative effects on control and pleasure for consumers who had a choice to enter a retail setting than for consumers who had no choice.

Translated to the current context, these findings suggest that giving users a choice (via personalization) over how they will be addressed by a Web-based app, or what the app looks like, may increase feelings of control and, thereby, well-being. Moreover, giving people active control may transform otherwise passive patients at the mercy of hostile technology into active citizens responsible for their own well-being.

Personalization at the content level has been used in Web-based interventions only sparsely. An example is the study of Andersson et al [42] where users of a Web-based treatment of anxiety disorders could choose which modules they wanted to engage in.

### Design Approach II: Ambient Information

We live in a world of continuous information overflow. A reasonable part of the information we receive is aimed to influence us in some way. Guadagno and Cialdini [43] report how a colleague counted over 500 influence appeals over an hour. Due to the large amount of (influential) information that reaches us during our busy daily routines, we can easily miss out on the information that we *did* want to notice. Not overburdening users and spreading feedback through multiple modalities reduces cognitive workload, rendering interactions with health interventions more pleasurable (P in PERMA) and effective, triggering feelings of accomplishment (A in PERMA). A huge drawback of Web-based interventions in our current information-dense world is that they are for the most part not visible to their users. People may, therefore, easily forget to go online to use the intervention. Moreover, when they do think about going online, they may want to ignore this thought because they did not enjoy working with the intervention before and may not feel the *need* to go online. The design of a Web-based intervention should accommodate for this *need* or *desire*. Designers of Web-based interventions should consider the information overflow that their users will inevitably face, and think carefully about what information they have to give, and at what point or in which context. At the same time, users may need status/progress information that is at hand or in sight in order to evoke a sense of urgency to use the Web-based intervention. Providing information in different places or providing feedback through other modalities than the often-used visual modality could accommodate for this.

Designers have explored both of these approaches in research on the design and effect of ambient information and in explorations on peripheral interaction. The concept of peripheral interaction deals with how technology and interactions that people have with technology is integrated throughout our everyday activities. Peripheral interaction entails that the attention that we pay to technology can at times take place in the periphery of attention. Bakker et al [44] explored how different modalities can be used for peripheral interaction and used both physical (ie, tangible) interaction and auditory displays. The concept of peripheral interaction is similar to approaches that have been used earlier in the design of so-called awareness systems. Such systems have been targeted at displaying one type of information—that of presence of other people at another location—in isolation on a physical object, making this information much more accessible and prominent. An example of such a system is the SnowGlobe [45] that displays movement of a remote user by glowing brighter and that also offers direct opportunities for interaction.

**Figure 5:**
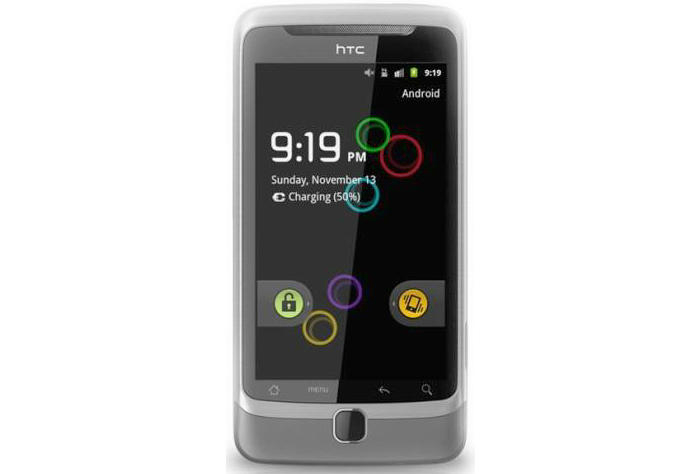
The mobile phone wallpaper, Bouncers, by Terence Nelson.

In their work on lifestyle behavior change technologies, Consolvo et al [46] define four design strategies and argue that presenting "abstract" information rather than specific information would have a positive effect on the effectiveness of persuasive systems. This is in line with what Nelson [47] incorporated in his design of Bouncers (see Figure 5). Bouncers is a wallpaper on mobile phones of a group of friends that visualizes everyone’s activity through moving circles. In this abstract way, it tells its users about their movements in relation to that of their friends.

As another example of more abstract information display, Ham and Midden [48] compared reactions toward factual (ie, numeric) information and ambient information—light changing color—in the case of information about energy consumption and argue that giving ambient (ie, less specific) information can be more influential because it requires less cognitive capacity to process this information.

In summary, the experience of Web-based interventions can benefit from using different ways to communicate information. Depending on the situation someone is in, and on the importance of the information, designers can choose whether specific or more abstract information is the better choice, and can decide on the specific location of the information (ie, within the Web-based interventions or through a different device or location).

### Design Approach III: Use of Metaphors

One of the key challenges in designing Web-based apps is how to give shape in words and visualizations to challenges, goals, and feedback provided during interaction in order to provide a meaningful and engaging "story" (M and E in PERMA). In many traditional apps, emphasis is on concrete textual input (ie, instructions or numbers signifying scores or tasks left to accomplish) or concrete images (eg, wallpapers or avatars). However, in addition to such concrete elements, much of everyday thought and experience is inherently metaphorical in nature as originally demonstrated by Lakoff and Johnson in their first joint effort, Metaphors We Live By [49]. The central claim therein was that the way we think about abstract concepts, such as accomplishment, challenge, and perseverance, are metaphorical in nature and are rooted in everyday bodily experiences [49-51]. Of special relevance to the current undertaking was the finding that these embodied metaphors are by no means restricted to language use, but are highly "visual" in nature, as demonstrated by Forceville [52].

For instance, in metaphorical expressions such as "we’ve made it to the top," "we have a long way to go," and "she was keeping me at a distance," abstract meanings (ie, achievement, lack of communication, psychological support) are talked about in terms of visual-spatial patterns (ie, "rising to the top" or "being close or far away from an end goal"). Such couplings are embodied because they are grounded in everyday bodily interactions in and with our environments.

With respect to object perception, Van Rompay et al [53] showed that verticality or "relative height" is not only the basis of figurative expressions conveying dominance (eg, "He’s on top of his game"), but that it also steers meaning perception in design. For instance, in the latter study, everyday products of great vertical size were perceived as more dominant, proud, and impressive compared to products of lesser height.

Furthermore, recent studies have demonstrated that metaphor use or having people behave in line with employed metaphors may also impact feelings and behaviors [50,54,55]. For instance, Gibbs [50] recently showed that a successful relationship metaphor (eg, "Your relationship was moving along in a good direction") prompted people to walk further and longer afterward compared to when they were cued with an unsuccessful relationship metaphor (eg, a metaphor stressing thwarted progress along the road). Another study stressing embodied features of motivation and decision making showed that merely watching a series of squares expanding, versus contracting, prompted participants to feel more self-confident and capable of self-actualization [55].

In design research, various authors have explored metaphor use in relation to affect product experience [56,57]. For instance, Ludden et al [57] showed that product designers can create products incorporating metaphors that people understand by making mind maps revealing common associations from source to target, such that motivating aspects of a source domain (eg, excitement of a journey) can be transferred to the target domain (ie, the product).

Taken together, these examples and insights suggest that employing metaphors in Web-based apps may not just be fun or trivial, but that it may actually contribute to adherence in the long run by creating meaning and fostering engagement. Indeed, metaphors have been used for the design of mobile apps that seek to motivate people toward certain types of behavior. A well-known example in literature on persuasive design is the design of a flourishing garden that was used by Consolvo et al [58] as a metaphor for a flourishing (ie, active and satisfying) life.

In summary, depending on the type of app and the goals set out for the app, different types of metaphorical mappings may be drawn upon that may become ever more influential as we move from static, to dynamic, to fully interactive apps in the years to come. Not only can such visual metaphors enhance fun and engagement (positive emotions, P), they may also render goals that people set out for, and make the activities they undertake to achieve them, more meaningful and worthwhile, precisely because they connect to people’s intuitive understanding of the world around them.

**Figure 6:**
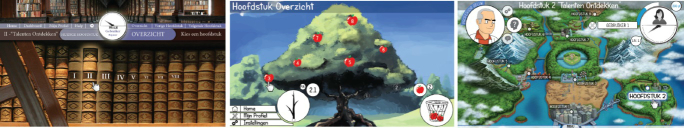
Three concept designs from the Web-based intervention, This Is Your Life! Concepts: library, tree of happiness, and journey on a map.

A nice example of an endeavor to use a metaphor in a Web-based intervention context is the intervention, This Is Your Life!, that was based on an existing positive psychology intervention [59], and designed for a specific target group: teachers at a primary school (see Ludden et al [60] for a full report of the design process). The designers of the interactive part of the intervention worked in close collaboration with the psychologists who designed the content of the intervention, and in close interaction with the proposed target group. A set of three concept designs was made in which different types of source domains (ie, a library, tree, and journey, respectively) were used (see Figure 6).

**Figure 7:**
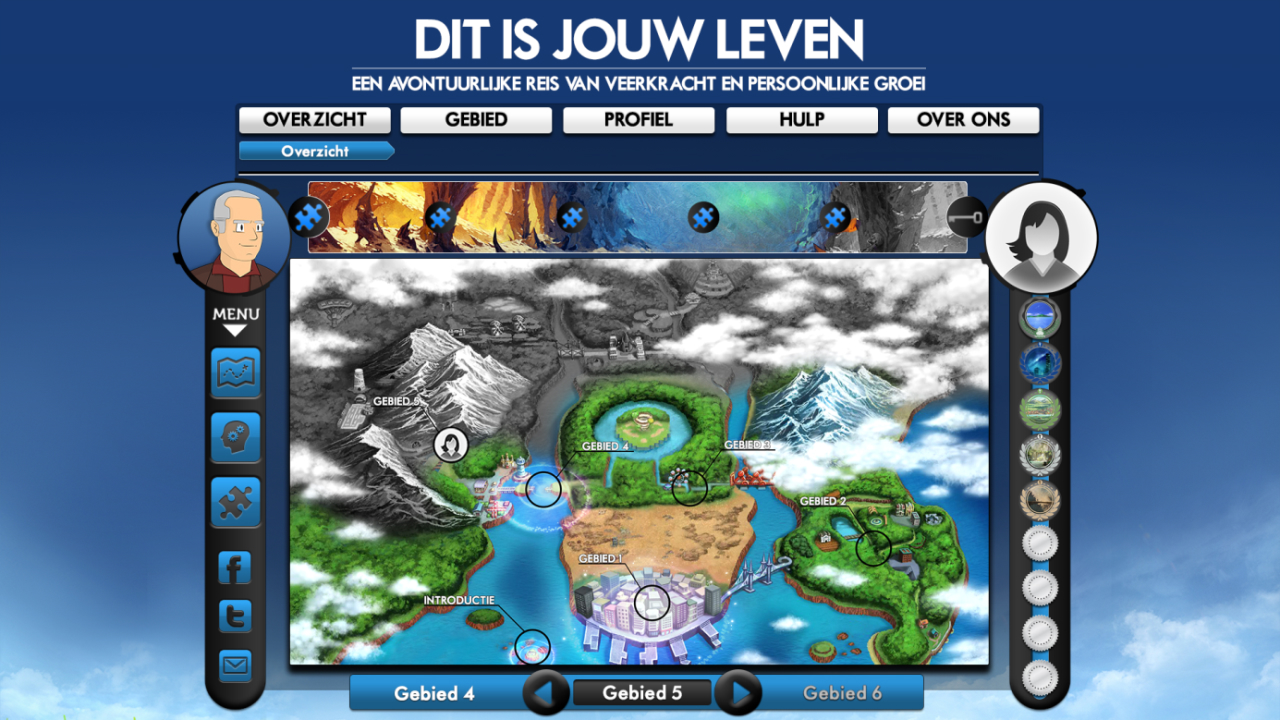
Final prototype of the Web-based intervention, This Is Your Life!

The concept of the intervention presented as a journey on a map—favored by all 8 participants in a focus group—was further developed into a working prototype (see Figure 7). For the final prototype, the metaphor of the journey was further developed into details of the intervention. For example, the typical terminology from training or school-like activities (ie, lessons, exercises, chapters) was changed into terminology that was more relevant to the metaphor of the journey. For instance, *chapters* are *locations* on the map and each location has *challenges* which are the *exercises* of the training. When a user completes the challenges for a specific location, he or she can get the "key" to the next location. Locations also have names that are related to the "life is a journey" metaphor, such as "The island of broken dreams" and "The river of flow."

From short, individual, evaluative sessions with primary school teachers, the developers of this intervention found that the metaphor that was used for the design of the intervention was seen as motivating and stimulating by users.

## Discussion

### The Role of Design(ers)

We have argued that designers of technology for well-being could stimulate well-being in two ways: by designing a positive experience during use and by developing persuasive effective interventions that positively influence adherence, leading to a higher level of overall well-being for the user. To specify how design can contribute, we have introduced three approaches that have recently gained particular attention in design research. In doing so, we have tried to broaden the discussion on the role of design in the development of Web-based interventions and eHealth in general. So far, this discussion has been mainly focused on the need to involve users and usability experts in the design process [61,62], as well as on transferring knowledge from behavioral sciences about persuasion techniques [63]. This has in most cases lead to detailed "design guidelines" about the organization and specific features of eHealth systems, factors that are, of course, of influence on how the interventions will be experienced. However, design guidelines that are more focused on the different ways in which these factors can eventually take shape and how this can have an effect on the experience of the user have so far mainly been neglected. That said, next to the three approaches introduced here, various other design approaches could also contribute to one of the elements of PERMA. We have chosen the three discussed here because they seem to be prevalent choices in recent design research in the eHealth domain. Inspiration for other approaches could, for example, be found in literature on how to apply elements of gaming in a therapy context [64]. However, the best way to ensure that all aspects contribute to a desired experience for an eHealth intervention is to facilitate cooperation between designers of the content of this technology (eg, the therapists developing health interventions), designers of the system (eg, human-computer interaction [HCI] developers), and designers of the form of the technology (eg, interaction designers or product designers) from an early stage of development, and discuss which design features and organization can enrich the presentation of the content. Together, this team of designers can reflect on questions such as “What seem to be the most promising new approaches/theories/tools?” “How will we apply these to practice?” “What is the future of Web-based health interventions?” and “How to incorporate more interactive and persuasive features?” This last question is also reflected in one of the principles of the holistic framework to improve the uptake and impact of eHealth technologies as described by van Gemert-Pijnen et al [64]. As we have argued and illustrated throughout this paper, involving users at an early stage of the design process can certainly be a valuable way to make informed design decisions on which persuasive/motivational elements to use.

### Future Research

In this paper, three approaches that have gained particular attention in design research were introduced and their possibilities to serve the design of Web-based interventions were discussed. Clearly, follow-up research is needed to further pinpoint the shortcomings in the current design of Web-based interventions, to test whether the approaches discussed indeed are effective, and to suggest how they may be applied in ever more advanced and interactive technologies.

For example, application possibilities of the design tools discussed here may also lie in third generation eHealth interventions. In this context, design can play an important role in, for example, the way feedback on behavior is offered to people through mobile and/or wearable coaching systems. Next to this, as Web-based apps or games are becoming more interactive—think, for instance, of serious gaming apps making use of a Wii or PlayStation Move controller—means for enriched metaphor portrayal are growing all the time. Hence, in addition to watching expanding stimuli (eg, expanding circles on a water surface) and feeling more self-confident—compare with Ludden et al [57]—such interactive game controllers may also prompt people to take in specific postures. Hence, when cultivation of open-mindedness is at stake, people may be prompted to take in an expansive, as opposed to a contracted, posture. This is again in line with linguistic expressions in which openness and intellectual growth are coupled, for example, "open up to others," "that blew my mind," and "her world is so small." Interestingly, the latter relationship between taking in an expansive posture and feeling more confident was recently demonstrated by Carney et al [54]. Specifically, they showed that people taking in an expansive bodily posture displayed more self-confidence and were less likely to seek compromise in a negotiation task. Hence, future research could address means by which "priming" characteristics, such as open-mindedness and self-confidence, may contribute to adherence in the long run.

As for reach and tailored communication with specific target groups, it is important to keep in mind that different target groups may react differently depending on metaphor choice and personalization options. Recently, Halko and Kientz [35] showed that the type of appeal exerted by persuasive technologies may indeed sort different effects dependent on target group personalities, with a more authoritarian style of interaction, for instance, faring better with some target groups than others.

As for personalization and feelings of control, numerous studies have likewise shown that people vary in the extent to which they seek and appreciate control over events in their lives [65,66]. People high in desire for control generally seek influence over others and desire control over events, and hence react with a strong (negative) emotional response when opportunities for control are lacking. In the online context, this may thus lead to nonadherence and dropout. People low in desire for control, on the other hand, are less likely to react with negative affect when faced with a perceived inability to control events, and may appreciate it when decisions are made by others.

Prior to metaphor selections and making decisions on how specific feedback information should be, on whether to incorporate nonvisual information, and on whether or not to incorporate personalization options in Web apps, insight into the values and needs of different target groups is a prerequisite. In the online context, such insights may come in the form of short intake questionnaires, based on which such decisions can be made. In conclusion, using design research as a source of inspiration for the design of Web-based interventions, as outlined in this paper, is a promising means to optimize the reach of, and adherence to, such interventions. Moreover, cooperation between different types of designers of interventions is, next to involving the envisioned users of an intervention, essential to come to a design that incorporates all aspects (ie, content, form, organization) that can contribute to a desired experience for the end user. Moreover, this cooperation should start in early phases of the development, not after a "who, what, and why" has been proposed for an intervention. Early involvement of all types of designers will allow for more innovative designs in which the different elements of the intervention can be experienced in an integrated way.

## Abbreviations

COPD: chronic obstructive pulmonary disease
HCI: human-computer interaction
PERMA: positive emotions, engagement, positive relationships, meaning, and accomplishment
